# Long-Term Efficacy and Safety of Repeated Intravescial OnabotulinumtoxinA Injections Plus Hydrodistention in the Treatment of Interstitial Cystitis/Bladder Pain Syndrome

**DOI:** 10.3390/toxins7104283

**Published:** 2015-10-22

**Authors:** Cheng-Ling Lee, Hann-Chorng Kuo

**Affiliations:** Department of Urology, Buddhist Tzu Chi General Hospital and Tzu Chi University, 707, Section 3, Chung Yang Road, Hualien 97002, Taiwan; E-Mail: leecl@hotmail.com

**Keywords:** follow-up, interstitial cystitis/bladder pain syndrome, onabotulinumtoxinA

## Abstract

Intravesical onabotulinumtoxinA (BoNT-A) injection can relieve symptoms of interstitial cystitis/bladder pain syndrome (IC/BPS), but lacks sustainability. Repeated injections have been shown to provide a superior outcome to a single injection, but data on long-term efficacy and safety is limited. In this prospective study, we enrolled patients with refractory IC/BPS, and treated them with 100 U of BoNT-A injection plus hydrodistention followed by repeated injections every six months for up to two years or until the patient wished to discontinue. A “top-up” dose was offered after the fourth injection. Of these 104 participants, 56.7% completed four BoNT-A injections and 34% voluntarily received the fifth injection due to exacerbated IC symptoms. With a follow-up period of up to 79 months, O’Leary-Sant symptom and problem indexes (ICSI, ICPI, OSS), pain visual analogue scale (VAS) functional bladder capacity, frequency episodes, and global response assessment (GRA) all showed significant improvement (*p* < 0.0001). Those who received repeated injections had a better success rate during the long-term follow-up period. The incidence of adverse events did not rise with the increasing number of BoNT-A injections. A higher pre-treatment ICSI and ICPI score was predictive for successful response to repeated intravesical BoNT-A injections plus hydrodistention.

## 1. Introduction

Bladder pain, urinary frequency, urgency, and sterile urine resemble the characteristics of interstitial cystitis/bladder pain syndrome (IC/BPS). It is a chronic and debilitating urologic condition that affects millions of people worldwide. Despite the fact that the exact cause of IC/BPS remains obscure, the underlying pathophysiology may involve a combination of chronic inflammation, mast cell activation, autoimmune response, neurological dysregulation, and urothelial dysfunction [1]. A variety of treatment modalities have been proposed, but very few or none have been successful in eradicating bladder pain and increasing bladder capacity [2]. Oral medications such as amitriptyline, cyclosporine A, or pentosan polysulphate have not been shown to provide long-term efficacy [1]. Intravesical instillation of hyaluronic acid is believed to protect leaky urothelium from irritants; however, a high symptom recurrence rate was reported [3].

Botulinum toxin is a potent neurotoxin produced by *Clostridum botulinum*. It consists of a heavy chain and a light chain linked together by a single disulfide bond. Once administrated, the heavy chain binds to cholinergic nerve terminals, and after internalization, the light chain binds with soluble *N*-ethylmaleimide-sensitive factor attachment protein receptor complex and cleaves synaptosomal-associated proteins of 25 kDa, which inhibits synaptic exocytosis and disables neural transmission [4,5].

While not approved by Food and Drug Administration in the U.S. for IC/BPS, onabotulinumtoxinA (BoNT-A) has been reported to treat a list of urologic conditions successfully since 1988, including bladder outlet obstruction, detrusor over-activity (both idiopathic and neurogenic), and detrusor sphincter dyssynergia [6]. Recently, randomized, controlled trials and prospective cohort studies have demonstrated BoNT-A as a promising treatment for IC/BPS [7,8]. It is now considered an effective and safe option for those who have failed behavioral and oral therapy prior to undertaking major surgery [9]. However, the efficacy of a single dose of BoNT-A diminishes with time and repeated injections may be required to sustain the clinical effect [10]. The purpose of this study was to evaluate the long-term effectiveness and safety of repeated intravesical BoNTA injections plus hydrodistention in IC/BPS patients unresponsive to conventional therapy.

## 2. Results

A total of 104 patients (16 men and 88 women, mean age 46.7 ± 14.8 and 48.5 ± 11.9 years, respectively) entered this study and received the initial BoNT-A injection. Table 1 outlines the therapeutic effects at three and six months after the initial BoNT-A injection. At six months, the overall OSS (23.7 ± 6.1 *vs*. 16.6 ± 8.9, *p* < 0.0001), VAS (5.2 ± 2.4 *vs*. 3.5 ± 2.5, *p* < 0.0001), FBC (129.1 ± 75.0 *vs*. 177.7 ± 85.0, *p* < 0.0001) and daytime frequency episodes (15.3 ± 7.7 *vs*. 11.3 ± 6.3, *p* < 0.0001) all showed significant improvement after one single injection. GRA was also improved (1.31 ± 0.97, *p* < 0.0001).

**Table 1:** Changes of parameters at three and six months after the initial BoNT-A injection in 104 patients.

BoNT-A (*N* = 104)	Baseline	3 M	6 M	*p* value
ICSI	12.3 ± 3.4	9.1 ± 4.5	8.8 ± 4.6	<0.0001
ICPI	11.4 ± 3.0	7.7 ± 4.4	7.8 ± 4.5	<0.0001
OSS (ICSI+ICPI)	23.7 ± 6.1	16.8 ± 8.7	16.6 ± 8.9	<0.0001
VAS	5.2 ± 2.4	3.4 ± 2.5	3.5 ± 2.5	<0.0001
FBC	129.1 ± 75.0	180.6 ± 89.1	177.7 ± 85.0	<0.0001
Frequency	15.3 ± 7.7	11.9 ± 6.5	11.3 ± 6.3	<0.0001
Noturia	4.7 ± 4.7	3.5 ± 3.8	3.5 ± 3.8	<0.0001
Qmax	12.7 ± 5.4	13.9 ± 6.0	14.6 ± 6.1	0.018
Volume	243.5 ± 110.6	238.7 ± 121.5	238.6 ± 123.2	0.802
PVR	39.9 ± 94.9	47.9 ± 77.4	48.1 ± 78.2	0.260
CBC	276.7 ± 108.8	285.9 ± 137.9	286.5 ± 143.9	0.510
GRA	0	1.26 ± 0.98	1.31 ± 0.97	<0.0001

ICSI: interstitial cystitis symptom index, ICPI: interstitial cystitis problem index, OSS: O’Leary-Saint symptom score, VAS: visual analog score, FBC: functional bladder capacity, Qmax: maximum flow rate, PVR: postvoid residual volume, CBC: cystometric bladder capacity, GRA: global response assessment.

**Table 2:** Changes of parameters in 59 patients receiving all four BoNT-A injections.

BoNT-A (*N* = 59)	BoNT-A(1) Baseline	BoNT-A(2) Baseline	BoNT-A(3) Baseline	BoNT-A(4) Baseline	*p* value
ICSI	12.6 ± 3.5	8.9 ± 4.5	8.7 ± 3.9	8.3 ± 4.2	<0.0001
ICPI	11.9 ± 2.9	8.2 ± 4.6	7.9 ± 4.3	6.8 ± 4.9	<0.0001
OSS	24.6 ± 6.1	17.1 ± 8.8	16.5 ± 8.1	15.2 ± 8.9	<0.0001
VAS	5.4 ± 2.2	3.6 ± 2.2	3.3 ± 2.4	2.9 ± 2.3	<0.0001
FBC	133.5 ± 74.0	172.2 ± 83.8	205.8 ± 93.9	226.9 ± 108.8	<0.0001
Frequency	15.2 ± 7.1	10.8 ± 5.6	10.9 ± 5.8	10.3 ± 5.3	<0.0001
Noturia	4.1 ± 2.9	3.3 ± 2.8	2.9 ± 2.2	3.2 ± 2.5	0.044
Qmax	13.9 ± 4.7	13.3 ± 6.0	13.2 ± 4.8	12.9 ± 5.5	0.830
Volume	260.3 ± 101.6	277.2 ± 119.9	293.7 ± 127.8	282.1 ± 147.7	0.401
PVR	17.1 ± 38.1	42.4 ± 77.9	47.8 ± 84.7	64.1 ± 114.2	0.015
CBC	272.9 ± 110.7	316.0 ± 107.1	334.3 ± 109.4	345.2 ± 149.4	0.006
MBC	677.7 ± 217.4	745.0 ± 205.9	728.6 ± 222.2	756.4 ± 192.2	0.013
GRA	0	1.3 ± 1.1	1.5 ± 0.9	1.8 ± 1.1	<0.0001
Glomerulation	1.7 ± 1.0	1.5 ± 0.9	1.3 ± 0.9	1.3 ± 0.9	0.006

Those patients were followed up to 79 months. Ninety patients decided to undergo a second dose of BoNT-A injections, 72 patients received the third injection, and 59 patients completed the four courses of BoNT-A injections. Table 2 lists the variables included in the GRA, symptom scores, VAS, voiding diary, uroflowmetry, and maximal bladder capacity (MBC) at the baseline of each injection time point in those who received four BoNT-A injections. The GRA has a trend to increase with the increasing injection number. The measured variables at all time points in patients who received four BoNT-A injections revealed a marked improvement in OSS (24.6 ± 6.1 *vs*. 15.2 ± 8.9, *p* < 0.0001), VAS (5.4 ± 2.2 *vs*. 2.9 ± 2.3, *p* < 0.0001), FBC (133.5 ± 74.0 *vs*. 226.9 ± 108.8, *p* < 0.0001), and daytime frequency (15.2 ± 7.1 *vs*. 10.3 ± 5.3, *p* < 0.0001). Although the glomerulation grade showed significant improvement (1.7 ± 1.0 *vs*. 1.3 ± 0.9, *p* = 0.006), MBC (677.7 ± 217.4 *vs*. 756.4 ± 192.2, *p* = 0.013) did not improve significantly after four BoNT-A injections.

**Figure 1:**
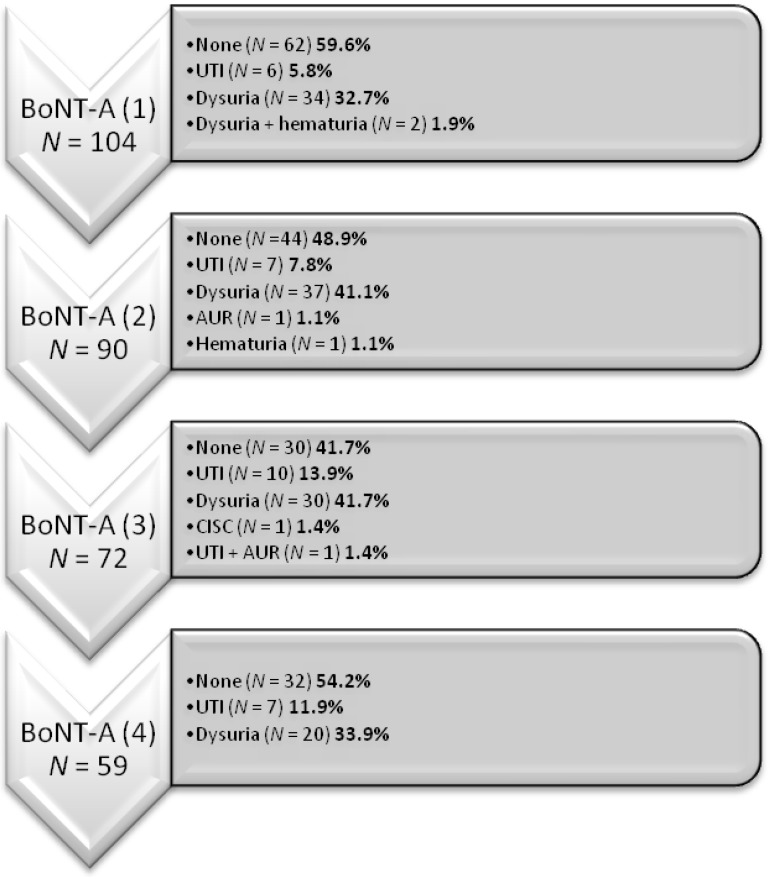
Documented adverse events in each treatment time point.

The adverse events at each treatment time point are shown in Figure 1. Dysuria is the most frequently reported symptom after each BoNT-A injection (range from 32.7% to 41.7%). Urinary tract infection (UTI) developed in 5.9% to 13.9% of patients after each BoNT-A treatment. There were two acute urinary retention episodes that occurred and one patient temporarily required clean intermittent self-catheterization. The occurrence of adverse events did not increase with the increasing number of BoNT-A injections.

**Figure 2:**
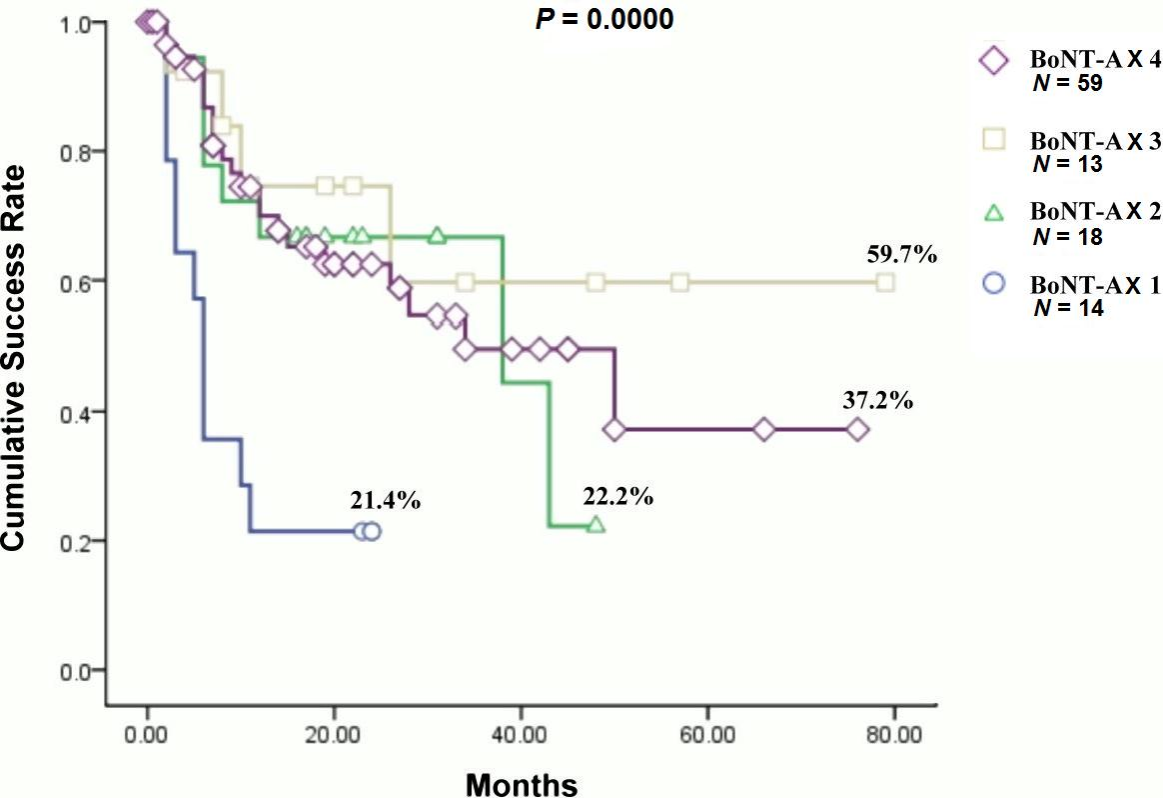
The cumulative success rate of the patients who received one to four BoNT-A injections.

At six months after the fourth BoNT-A injection, 37 (62.7%) of 59 patients had improvements in GRA by two points compared to their baseline conditions. Figure 2 shows a long-term success rate up to 79 months in patients who received repeated BoNT-A injections (*p* = 0.000). Kaplan-Meier survival curves revealed significantly better results in those who had three repeated injections (59.7%) and four injections (37.2%). After the third injections, most patients with a good response decided not to proceed further.

We also investigated the possible predictive factor for a successful outcome (GRA ≥ 2). We found there is a positive correlation between pre-treatment ICSI and ICPI scores with a successful outcome following repeated intravesical BoNT-A injections (Table 3).

**Table 3:** Correlation between baseline parameters and a GRA ≥ 2 in patients who received all four treatments.

Parameters	ICSI	ICPI	VAS	FBC	Frequency	Nocturia	Qmax	PVR
ICSI	1	0.861	0.278	−0.197	0.369	0.361	−0.118	−0.184
		0.000	0.200	0.368	0.083	0.091	0.611	0.412
ICPI	0.861	1	0.157	−0.226	0.387	0.226	−0.110	0.030
	0.000		0.473	0.301	0.068	0.300	0.636	0.894
VAS	0.278	0.157	1	−0.273	0.080	0.339	−0.089	−0.113
	0.200	0.473		0.207	0.716	0.114	0.702	0.617
FBC	−0.197	−0.226	−0.273	1	−0.482	−0.300	0.530	−0.013
	0.368	0.301	0.207		0.020	0.165	0.013	0.953
Frequency	0.369	0.387	0.080	−0.482	1	0.236	−0.540	0.136
	0.083	0.068	0.716	0.020		0.277	0.011	0.545
Nocturia	0.361	0.226	0.339	−0.300	0.236	1	0.006	0.004
	0.091	0.300	0.114	0.165	0.277		0.979	0.988
Qmax	−0.118	−0.110	−0.089	0.530	−0.540	0.006	1	−0.022
	0.611	0.636	0.702	0.013	0.011	0.979		0.926
PVR	−0.184	0.030	−0.113	−0.013	0.136	0.004	−0.022	1
	0.412	0.894	0.617	0.953	0.545	0.988	0.926	

Among the 59 patients receiving four BoNT-A injections, 20 (34%) decided to receive the fifth BoNT-A injection due to IC symptoms exacerbated at five to 28 (mean 13.7 ± 6.8) months after the foruth injection, 30 (51%) chose watchful waiting because the symptoms remained unchanged with a follow-up period of five to 79 (mean 25.3 ± 18.2) months, seven patients (12%) converted to augmentation enterocystoplasty (*n* = 3) or electrofuguration (*n* = 4) due to Hunner’s lesion and contracted bladder, and the other two withdrew from the study in poor bladder condition.

## 3. Discussion

The management of IC/BPS is mainly directed toward symptomatic relief. The application of BoNT-A has been shown to reduce bladder pain and impaired bladder sensation, and decrease chronic inflammation in the central nervous system in the animal model [11]. In this prospective study, we observed a significant improvement in both subjective symptoms and functional parameters in refractory IC/BPS after repeated intravesical injections of BoNT-A plus hydrodistention. It is well tolerated with no marked difference in reported adverse events as the number of injections increases on a long-term follow-up basis.

Our current knowledge regarding BoNT-A in the treatment of IC/BPS was built upon a few clinical trials in the early 2000s. Intradetrusor BoNT-A injection was reported to have an antinociceptive effect five to seven days after it was administrated and its effect lasted a mean of 3.72 months [12]. At the one- and three-month follow-up study, Giannantoni *et al*. published an initial subjective improvement in 86.67% of IC/BPS patients, but its therapeutic effect declined 60% within five months and none had any effect at 12 months after the initial treatment [10,13]. With a larger study population, we have also shown a similar outcome of intravesical BoNT-A injection. It was capable of reducing ICSI and ICPI scores, increasing functional bladder capacity, decreasing frequency and nocturia, and improving GRA at three and six months. As attested by animal and human trials, this favorable outcome may have contributed by inhibiting the release of acetylcholine at neuromuscular junctions, in turn causing muscle relaxation. In addition, by regulating neuromodulators (*i.e.*, substance P), BoNT-A is believed to modify pain perception, vasodilatation, and neurogenic inflammation [4]. In conjunction with hydrodistension, BoNT-A was shown to have a synergic effect on pain relief and increase bladder capacity [14].

However, the efficacy of a single dose of BoNT-A diminishes with time and repeated injections may be required to sustain the clinical effect [9]. In our previous pilot study, we observed a better clinical outcome in patients who were treated with repeated intravesical BoNT-A injections as opposed to a single injection [15]. A two-year follow-up study conducted by Pinto *et al*. revealed a high percentage of patients were willing and demanded repeated BoNT-A injections because of marked improvement in their symptoms [16]. In this current study, we unveiled a better symptomatic relief and a longer therapeutic duration in patients receiving repeated injections. Besides, it is not uncommon for patients to have and benefit from a “top-up” intravesical BoNT-A injection when their symptoms are exacerbated at long term follow-up. A one-off intravesical BoNT-A injection relieves symptoms of IC/BPS temporarily, and an inadequate anti-apoptotic and anti-inflammatory effect is likely to cause failure in long-term efficacy [17,18]. Immunohistochemical evidence indicates that repeated BoNT-A injections can significantly suppress apoptotic signaling proteins Bax, p-p38, and mast cell activity tryptase expression, and further reduce the expression of sensory receptors (M2, M3, P2X2, and P2X3) [19,20]. Taken together, this reduction could lead to a profound anti-inflammatory effect and peripheral desensitization, resulting in longer and greater success in the treatment of IC/BPS.

As previously discussed, apart from encouraging improvement in subjective symptoms, the degree of glomerulation was also significantly lower in patients who received repeated BoNT-A injections [15]. Vascular endothelial growth factor (VEGF) plays a role in stimulating vasculogenesis and angiogenesis during cellular injury. Kiuchi *et al*. found that VEGF over-expression is highly associated with the degree of glomerulation [21]. Increased VEGF was associated with bladder inflammation and smaller functional bladder capacity in patients with IC/BPS and decreased after repeated BoNT-A injections and hydrodistention [22]. We suggested that the glomerulation and increased vascular permeability resulting from inflammatory stimulation in IC/BPS and that BoNT-A could somehow attenuate the expression of angiogenic markers and inflammation after repeated injections [15,19,23].

While BoNT-A has been used widely since the 1970s, there are still concerns about long-term safety and resistance with its repeated administration [4]. A review of previous investigations of BoNT-A on urologic conditions shows variable results. Gottsch reported no systemic or local complications after injecting 50 U BoNT-A into the bladder neck [24]. On the contrary, we reported only 4.9% of patients had UTIs, 28.4% had dysuria, and 2.5% had hematuria after receiving 100 U of BoNT-A in refractory IC/BPS. Although occasional unwanted urinary symptoms were reported, the occurrence of adverse events did not grow with an increase in the number of BoNT-A injections (*p =* 0.235) [14]. The conclusion on the risk of developing bladder fibrosis following frequent treatment with BoNT-A has not been drawn [25]. In the present study, most of the adverse events were self-limited and rarely required interventions.

The risk of developing antibody-induced therapy failure appears to be low and depends on several variables, including patient-related factors, the dose administered, the immunologic quality of the preparation, and the interval between injections, *etc*. [26]. In this study, we examined the potential predictive factors for successful repeated intravesical BoNT-A injections in patients with refractory IC/BPS. There did not appear to be any links between age, initial VAS score, numbers of frequency/urgency episodes, or uroflowmetry parameters. Interestingly, we found there is a positive correlation between pre-treatment ICSI and ICPI scores and successful outcome following repeated intravesical BoNT-A injections. Based on this observation, we hypothesize that in refractory IC/BPS, patients with a higher pre-treatment OSS (ICSI and ICPI) score are most likely to benefit from repeated intravesical BoNT-A injections.

The main limitation of this study is the lack of a control arm. Due to ethical concern, it is unconscionable to use a placebo in such a specific, vulnerable patient group. Nevertheless, we demonstrated long-term effects on symptomatic improvement and objective increment in functional bladder capacity by repeated intravesical BoNT-A injections, without causing serious side effects.

## 4. Experimental Section

From July 2006, we first began our prospective study to follow consecutive patients diagnosed with refractory IC/BPS. A diagnosis of IC/PBS was established based on characteristic symptoms in accordance with the National Institute of Diabetes Digestive and Kidney Diseases and cystoscopic findings of glomerulations, petechia, mucosal fissure, or ulceration. At least one of the following medications has been tried for more than one year: oral pentosanpolysulphate, intravesical instillation of heparin, hyaluronic acid, or tricyclic antidepressant, but the symptoms remained unchanged or had relapsed.

The IC/BPS symptoms were assessed using the OSS including ICSI and ICPI. Special attention was drawn to bladder pain with daily activity. Pan intensity was scored using a 10-point VAS system (0 = no pain; 10 = worst pain) before and after treatment. All patients underwent a comprehensive video urodynamic study with a Urolab Janus 6 device (Life- Tech, Inc., Stafford, TX, USA) using a double lumen 6 Fr catheter conducted by one examiner in an identical manner. After the urodynamic study, 40 mL of 0.4 M KCl solution was infused slowly into the bladder and the test was regarded as positive when painful (increased VAS score ≥ 2) or urgency sensation was elicited compared to normal saline infusion during the study.

Intravesical injection of 100 U of BoNT-A (onabotulinumtoxinA, Allergan, Irvine, CA, USA) was injected followed by cystoscopic hydrodistention under intravenous general anesthesia. Each vial containing 100 U of BoNT-A was diluted with 10 mL of normal saline and delivered at 20 suburothelial locations. The needle was injected into the urothelium at the posterior and lateral walls of the bladder, using a 23-gauge needle and rigid cystoscopic injection instrument (22 Fr, Richard Wolf, Knittlingen, Germany). Although the practice of hydrodistention has not been standardized, it was reported that the maximal bladder capacity could be further increased after 10 min of distention. Prolonged hydrodistension (>30 min), on the other hand, could raise the risk of developing more anesthetic and urological complications [27,28]. We performed cystoscopic hydrodistention to an intravesical pressure of 80 cm of water for 15 min and the maximal bladder capacity (MBC) under hydrodistention was recorded.

When glomerulation, petechia, or mucosal fissure developed after bladder deflation, it was graded from none (0) to severe (4). Bladder biopsies were taken at four sites about 2 cm lateral and posterior to the ureteral orifice immediately after hydrodistention. The purpose of the bladder biopsy was to exclude the possibility of carcinoma *in situ*. A 14-Fr urethral Foley catheter was indwelled for one day after injection and an oral antibiotic was prescribed for seven days upon discharge. Patients were monitored in the outpatient clinic two weeks later and had a follow-up every three months. During each visit, a three-day voiding diary, symptom scores, pain VAS, and global response assessment (GRA) were reassessed. The largest voided volume in the three-day voiding diary was considered the measure of functional bladder capacity (FBC).

The primary end-point was the change in the sum of the ICSI and ICPI from baseline to six months after the initial treatment. Patients were requested to rate their bladder symptoms by GRA on a seven-point centered scale from markedly (−3), moderately (−2), and slightly worse (−1), no change (0), to slightly (+1), moderately (+2), and markedly improved (+3). Successful outcome is defined as those with moderate and marked improvement, whereas the rest were considered to have failed. Additionally, patients were informed of the possible complications associated with BoNT-A injections such as generalized muscle weakness, difficult urination, transient urinary retention, or urinary tract infection (UTI). This study was approved by the Institutional Review Board (TCGH100-06) and ethics committee of the Tzu-Chi General Hospital.

At six months after the initial BoNT-A injection, patients were questioned about their bladder condition. Repeat BoNT-A injection and hydrodistention were recommended six months after the initial treatment if patients felt a relapse of baseline symptoms or desired repeated treatment to achieve a better treatment outcome. The BoNT-A injection was repeated every six months up to four times or until patients declared their symptoms had significantly resolved or patients did not want repeated treatment because of lack of efficacy or adverse events. After the fourth injection, patients were followed up for as long as possible without any treatment. However, they could choose to have a fifth BoNT-A “top-up” injection on demand if their IC symptoms relapsed, or they could convert to another surgical modality or watchful waiting if symptoms remained unchanged. Videourodynamic study was performed again at six months after each BoNT-A treatment.

Continuous variables are presented as means ± standard deviations (SDs), and categorical data are presented as numbers and percentages (%). Statistical comparisons between the groups were tested using the chi-square test for categorical variables and the Wilcoxon rank sum test for continuous variables. Repeated measurement analysis was used for analysis of variables at different time points. Long-term successful results were compared using Kaplan-Meier analyses. Statistical assessments were considered significant when *p* < 0.05. Statistical analyses were performed using SPSS 15.0 statistical software (SPSS Inc., Chicago, IL, USA).

## 5. Conclusions

In this large long-term follow-up study, we have shown that repeated intravesical injections of BoNT-A are safe, effective, and sustainable in the treatment of IC/BPS patients who did not respond to conventional treatment.
